# Honokiol: A non-adipogenic PPARγ agonist from nature^☆^

**DOI:** 10.1016/j.bbagen.2013.06.021

**Published:** 2013-10

**Authors:** Atanas G. Atanasov, Jian N. Wang, Shi P. Gu, Jing Bu, Matthias P. Kramer, Lisa Baumgartner, Nanang Fakhrudin, Angela Ladurner, Clemens Malainer, Anna Vuorinen, Stefan M. Noha, Stefan Schwaiger, Judith M. Rollinger, Daniela Schuster, Hermann Stuppner, Verena M. Dirsch, Elke H. Heiss

**Affiliations:** a Department of Pharmacognosy, University of Vienna, Althanstrasse 14, 1090 Vienna, Austria; b Xi Yuan Hospital, China Academy of Chinese Medical Sciences, Beijing 100093, China; c Institute of Pharmacy/Pharmacognosy, Center of Molecular Biosciences, University of Innsbruck, Innrain 80-82, A-6020 Innsbruck, Austria; d Institute of Pharmacy/Pharmaceutical Chemistry, Center of Molecular Biosciences, University of Innsbruck, Innrain 80-82, A-6020 Innsbruck, Austria

**Keywords:** AMPK, AMP-activated kinase, ANOVA, analysis of variance, ATCC, American type culture collection, BADGE, bisphenol A diglycidyl ether, BMP, bone morphogenic protein 4, BSA, bovine serum albumin, CMCNa, sodium carboxymethyl cellulose, DMEM, Dulbecco's modified Eagle's medium, DMSO, dimethyl sulfoxide, EC_50_, effective concentration 50%, EGFP, enhanced green fluorescent protein, FCS, fetal calf serum, GST, glutathione-S-transferase, HPLC, high-performance liquid chromatography, IBMX, 3-isobutyl-1-methylxanthine, mTOR, mammalian target of rapamycin, NBS, newborn bovine serum, NF-κB, nuclear factor κB, NMR, nuclear magnetic resonance, PBS, phosphate buffered saline, PPARγ, peroxisome proliferator-activated receptor gamma, LBD, ligand-binding domain, MEF, mouse embryonic fibroblasts, NOX, NADPH-dependent oxidase, RXR, retinoic X receptor, SEM, standard error of the mean, SPF, specific pathogen free, SREBP, sterol regulatory element binding protein, TCM, traditional Chinese medicine, TLC, thin layer chromatography, TR-FRET, time-resolved fluorescence resonance energy transfer, Peroxisome proliferator-activated receptor, Natural product, Metabolic disease

## Abstract

**Background:**

Peroxisome proliferator-activated receptor gamma (PPARγ) agonists are clinically used to counteract hyperglycemia. However, so far experienced unwanted side effects, such as weight gain, promote the search for new PPARγ activators.

**Methods:**

We used a combination of in silico, in vitro, cell-based and in vivo models to identify and validate natural products as promising leads for partial novel PPARγ agonists.

**Results:**

The natural product honokiol from the traditional Chinese herbal drug Magnolia bark was in silico predicted to bind into the PPARγ ligand binding pocket as dimer. Honokiol indeed directly bound to purified PPARγ ligand-binding domain (LBD) and acted as partial agonist in a PPARγ-mediated luciferase reporter assay. Honokiol was then directly compared to the clinically used full agonist pioglitazone with regard to stimulation of glucose uptake in adipocytes as well as adipogenic differentiation in 3T3-L1 pre-adipocytes and mouse embryonic fibroblasts. While honokiol stimulated basal glucose uptake to a similar extent as pioglitazone, it did not induce adipogenesis in contrast to pioglitazone. In diabetic KKAy mice oral application of honokiol prevented hyperglycemia and suppressed weight gain.

**Conclusion:**

We identified honokiol as a partial non-adipogenic PPARγ agonist in vitro which prevented hyperglycemia and weight gain in vivo.

**General significance:**

This observed activity profile suggests honokiol as promising new pharmaceutical lead or dietary supplement to combat metabolic disease, and provides a molecular explanation for the use of Magnolia in traditional medicine.

## Highlights

• Honokiol is identified and characterized as novel partial PPARγ agonist from nature.
• In cell models honokiol increases glucose uptake but is not adipogenic.
• In KKAy diabetic mice it decreases blood glucose and suppresses weight gain.
• PPARγ agonism of honokiol may explain the use of Magnolia bark in traditional medicine.

## 1 Introduction

Sedentary lifestyle with low physical activity and high caloric intake promotes obesity, the metabolic syndrome, and type 2 diabetes, which pose a major risk for the individual's quality of life and a burden to the health care systems of industrialized societies. Peroxisome proliferator activated receptor gamma (PPARγ) agonists are clinically used to combat hyperglycemia common to these pathological conditions and to alleviate related comorbidities [1–3]. Generally, PPARs are nuclear receptors and ligand-dependent transcription factors which control lipid and glucose metabolism [4–7]. Upon ligand binding, PPARs form heterodimers with the retinoid X receptor (RXR), another nuclear receptor, and bind to response elements located in the promoter region of their target genes [8]. After recruitment of nuclear receptor coactivators [9,10] further chromatin rearrangement transcription is initiated [11]. From the three known subtypes of PPAR (*α*, *β*/*δ*, and *γ*), PPARγ is the best studied. It is expressed in adipose tissue, lung, large intestine, kidney, liver, heart, and macrophages [12]. The well-established important role in the regulation of glucose and lipid metabolism renders PPARγ a valid pharmacological target for combating metabolic diseases [13,14]. Currently available full PPARγ agonists represented by thiazolidinediones (e.g. pioglitazone) are clinically effective, but they have serious side- and off-target effects (e.g. weight gain or edema formation), urging the retrieval of new PPARγ agonists. Ligands acting as partial agonists induce submaximal receptor activation and have been demonstrated to retain beneficial anti-diabetic properties with reduced side effects. Despite lower activation of the receptor, they are still able to induce PPARγ target genes responsible for the anti-hyperglycemic and insulin sensitizing, but not for the unwanted PPARγ actions [15,16]. The reason for this is not entirely understood. However, it is conceivable that partial agonists may elicit a different conformation of the receptor–ligand complex that leads—by altered recruitment of transcriptional co-activators and repressors—to a more restricted set of expressed target genes compared to full agonists.

Using a computer-aided approach we have recently identified neolignans as a novel class of partial agonists occupying the PPARγ ligand-binding domain as dimers [17]. The hereby generated and optimized in silico tools now enabled us to identify and further characterize the neolignan honokiol, a major bioactive constituent of the traditional Chinese herbal drug Magnolia bark, as novel non-adipogenic partial PPARγ activator. The previously identified neolignans exhibit PPARγ-dependent adipogenic properties similar to pioglitazone [17]. In contrast, we show in this work that the newly identified PPARγ partial agonist honokiol does not trigger adipogenesis in two in vitro cell systems and prevents weight gain in the murine KKAy in vivo diabetes model, while retaining anti-hyperglycemic activity in vitro and in vivo.

## 2 Materials and methods

### 2.1 Chemicals, cell culture reagents, and plasmids

Fetal calf serum (FCS), newborn bovine serum (NBS) and Dulbecco's modified Eagle's medium (DMEM) were from Lonza (Basel, Switzerland). Pioglitazone used for all experiments except in vivo tests was from Molekula Ltd. (Shaftesbury, UK). All other chemicals were from Sigma-Aldrich (Vienna, Austria). The test compounds were dissolved in dimethyl sulfoxide (DMSO), divided into aliquots and kept frozen until use. In all experiments, DMSO was used as solvent control. For in vitro and cell-based assays, the final concentration of DMSO was kept ≤ 0.2%. The PPAR luciferase reporter plasmid (tk-PPREx3-luc) was a gift from Prof. Ronald M. Evans (Howard Hughes Medical Institute, La Jolla, CA) [18], the plasmid encoding enhanced green fluorescent protein (pEGFP-N1) was from Clontech (Mountain View, CA), and the plasmid encoding human PPARγ (pSG5-PL-hPPAR-γ1) was a gift from Prof. Walter Wahli and Prof. Beatrice Desvergne (Center for Integrative Genomics, University of Lausanne, Switzerland) [19].

### 2.2 Isolation of honokiol

880 g powdered bark of *Magnolia officinalis* (Plantasia, Oberndorf, Austria; lot: 710786) was exhaustively macerated with dichloromethane (8.0 l, 12 times) at room temperature, yielding 96.8 g crude extract. 80.0 g extract was separated by flash silica gel column chromatography (400 g silica gel 60, 40–63 μm, Merck, VWR, Darmstadt, Germany; 41 × 3.5 cm) using a petroleum ether–acetone gradient with an increasing amount of acetone. The eluate was collected in portions of 20 ml and analyzed by TLC. Comparable portions were combined to 18 fractions (A1–A18). Fraction A8 (5.761 g) was further separated by Sephadex LH-20-column chromatography (CC) using methanol as mobile phase. Fractions were monitored by TLC and combined according to their composition to 15 fractions (B1–B15). Fraction B8 (810 mg) was further purified by HSCCC with a mixture of PE–EtOAc–MeOH–H_2_0 1 + 0.5 + 1 + 0.5 (v/v/v/v) using the upper layer as mobile phase. HSCCC was set to the tail to head mode with 800 rpm and a flow rate of 1 ml/min. The eluate was collected in portions of 5 ml and combined to 18 fractions (C1–C18). Fraction C11 (eluted at 495–580 ml; 249 mg) was further purified by Sephadex LH-20-CC using a dichloromethane–acetone mixture (85 + 15, v/v) as mobile phase to yield 217.54 mg of honokiol in a purity (HPLC) of > 98%. Identity of the obtained compound was proven by comparison of 1- and 2D NMR-spectra with literature [20] and by LC–MS analysis. The obtained NMR- and LC–MS-data are provided as online supplement (supplemental Table S1 and supplemental Figs. S1–S4). The isolated honokiol was used for all in vitro bioassays.

### 2.3 PPARγ luciferase reporter gene transactivation

HEK-293 cells (ATCC, Manassas, VA) were grown in DMEM supplemented with 2 mM glutamine, 100 U/ml benzylpenicillin, 100 μg/ml streptomycin, and 10% FBS. The cells were seeded in 10-cm dishes at a density of 6 × 10^6^ cells/dish for 18 h, and then transfected by the calcium phosphate precipitation method with 4 μg of PPARγ expression plasmid, 4 μg of reporter plasmid (tk-PPREx3-luc), and 2 μg of pEGFP-N1 as internal control. Six hours later, the cells were reseeded in 96-well plates (5 × 10^4^ cells/well) in DMEM without phenol red with 5% charcoal-stripped FBS, glutamine and antibiotics. The cells were treated as indicated and incubated for 18 h. After cell lysis, the luminescence of the firefly luciferase and the fluorescence of EGFP were quantified on a GeniosPro plate reader (Tecan, Grödig, Austria). The luminescence signals were normalized to the EGFP-derived fluorescence, to account for differences in cell number or transfection efficiency. Neither pioglitazone nor honokiol interfered with the luciferase assay background determined upon transfection of the cells with tk-PPREx3-luc in the absence of PPARγ.

### 2.4 PPARγ competitive ligand binding

The LanthaScreen® time-resolved fluorescence resonance energy transfer (TR-FRET) PPARγ competitive binding assay (Invitrogen, Lofer, Austria) was performed using the manufacturer's protocol. The test compounds dissolved in DMSO or solvent vehicle (DMSO) alone were incubated together with the human PPARγ LBD tagged with GST, terbium-labeled anti-GST antibody and fluorescently labeled PPAR ligand (Fluormone Pan-PPAR Green). In this assay, the fluorescently labeled ligand is binding to the human PPARγ LBD, which brings it in close spatial proximity to the terbium-labeled anti-GST antibody. Excitation of the terbium at 340 nm results in energy transfer (FRET) and partial excitation of the fluorescent PPAR ligand, followed by emission at 520 nm. Test-compounds binding to the human PPARγ LBD are competing with the fluorescently labeled ligand and displacing it, resulting in a decrease of the FRET signal. The signal obtained at 520 nm is normalized to the signal obtained from the terbium emission at 495 nm; therefore, the decrease in the 520 nm/495 nm ratio is used as a measure for the ability of the tested compounds to bind to the human PPARγ LBD. Neither pioglitazone nor honokiol interfered with the background 520 nm/495 nm fluorescence in the absence of PPARγ LBD.

### 2.5 Induction of adipogenic differentiation

3T3-L1 preadipocytes were subcultivated in DMEM (containing 2 mM glutamine, 100 U/ml benzylpenicillin, 100 μg/ml streptomycin) plus 10% NBS. For differentiation experiments they were grown till confluency in 12-well plates. Two days later cells were treated with DMEM/10% FCS (negative control), DMEM/10% FCS/1 μg/ml insulin (*INS*, basal 3T3 differentiation medium), or *INS* medium + PPARγ agonists (1, 3 and 10 μM). An additional set of cells exposed to the highest concentration of the respective agonist was pretreated with the PPARγ antagonist BADGE (50 μM) for 30 min in order to assess the PPARγ-dependency of an observable lipid accumulation. All wells contained 0.2% DMSO. Medium was renewed every other day. After eight days, lipid accumulation (as measure of adipogenic differentiation) was determined by OilRed O staining. Solubilized dye was quantified at 550 nm. Experiments were performed at least three times.

Immortalized mouse embryonic fibroblasts (MEF) (kind gift of Prof. Thomas Kensler, University of Pittsburgh) were differentiated into adipocytes as described [21] with minor modifications. Cells were grown in DMEM/10% FCS till confluency (12-well plate). Two days later, cells were treated with DMEM/10% FCS (negative control) or basal MEF *diff* (differentiation) cocktail (1 μg/ml insulin/200 μM isobutylmethylxanthine (IBMX)/500 nM dexamethasone/25 ng/ml bone morphogenic protein (BMP) 4) in the presence and absence of PPARγ agonists (1–10 μM). An additional set of cells exposed to the highest concentration of the respective agonist was pretreated with the PPARγ antagonist BADGE (50 μM) for 30 min in order to assess the PPARγ-dependency of a potential lipid accumulation. All wells contained 0.2% DMSO. After three days, the medium was changed to DMEM/10% FCS for control cells and to DMEM/10% FCS/1 μg/ml ± PPARγ agonists (1–10 μM) for differentiating cells, respectively. After an overall differentiation time of nine days, lipid accumulation was determined by OilRed O staining (see supplemental Fig. S6). Solubilized dye was quantified at 550 nm. Experiments were performed at least three times.

### 2.6 OilRed O staining

Cells in plates were fixed with 10% formaldehyde for at least 1 h, washed with 60% isopropanol and dried, before filtered OilRed O solution (2% OilRed O in 60% isopropanol) was added for 10 min. After washing off the excessive dye with water, photos were taken, and bound dye was solubilized with equal volumes of 100% isopropanol per well and immediately photometrically quantified at 550 nm in a spectrophotometer (Tecan, Grödig, Austria).

### 2.7 2-Deoxy-d-(1H^3^)-glucose uptake assay in adipocytes

Superconfluent 3T3-L1 preadipocytes were differentiated into mature adipocytes for 10 days (2 days in DMEM (containing 2 mM glutamine and antibiotics) supplemented with 10% FCS, 500 μM IBMX, 1 μg/ml insulin and 500 nM dexamethasone followed by 2 days in DMEM/10% FCS/1 μg/ml insulin and 6 days in DMEM/10% FCS), and incubated with the test compounds or solvent vehicle for 24 h at 37 °C. Before the experiment, cells were incubated for 1 h in KRH buffer (50 mM HEPES, 136 mM NaCl, 23.5 mM KCl, 1.25 mM MgSO_4_, 1.25 mM CaCl_2_ and 0.1% BSA). After an additional KRH buffer change, cells were allowed to rest for 10 min, then glucose uptake was initiated by addition of 2-deoxy-d-glucose spiked with 2-deoxy-d-(1H^3^)-glucose (final concentrations of 0.1 mM and 0.45 μCi/ml). After 15 min the reaction was stopped by three rapid washes with ice-cold PBS. The glucose uptake rate was determined by liquid scintillation counting (PerkinElmer, Waltham, MA, USA) of cell lysates (0.05 N NaOH in PBS) and normalized to protein content assessed by the Bradford protein assay and uptake time (to obtain incorporated mol glucose per mg protein and minute).

### 2.8 Animal experiments

The KKAy mice represent a model for obesity and type 2 diabetes, with spontaneous development of hyperglycemia, hyperinsulinemia, glucose intolerance, and obesity [22,23]. KKAy mice (female, SPF grade, qualification license No. SCXK (Beijing) 2009-0004, certification No. 0237501, utilization license No. SYXK (Military) 2007-030), and C57B1/6J mice were all supplied by Beijing Hua Fu Kang Bio-technology Company Ltd. All animal tests were conducted with full compliance with local, national, and ethical principles and authority regulations. The KKAy mice were raised in a SPF experimental room, and served standard high fat diet (composed from sucrose, pork lard, egg yolk powder, and ordinary feed in ratio of 10:10:10:7; from Beijing Hua Fu Kang Bio-technology Company Ltd.) and freely available drinking water. Age-mated C57B1/6J mice were served ordinary feed and unrestricted drinking water. When the blood glucose of KKAy mice reached ≥ 13.9 mM (confirming the successful establishment of the diabetic model) the animals were separated into four groups (10 animals per group): (1.) “No treatment” group received through oral gavage administration sterilized water (0.1 ml/10 g body weight) once per day for 35 days; (2.) “Vehicle” group received alcoholic (0.5%) CMCNa (5%) solution (0.1 ml/10 g body weight); (3.) “Pioglitazone” group received through oral gavage pioglitazone hydrochloride solution (Santa Cruz Biotechnology, Heidelberg, Germany; 1 mg/ml alcoholic (0.5%)) CMCNa (5%) solution (0.1 ml/10 g body weight), once per day (10 mg/kg/d) for 35 days; (4.) “Honokiol” group received through oral gavage honokiol solution (Carbosynth, Compton, UK; 10 mg/ml alcoholic (0.5%)) CMCNa (5%) solution (0.1 ml/10 g body weight) once per day (the 100 mg/kg/d treatment dose was chosen because that concentration demonstrated to have a clear effect in preliminary experiments), for 35 days. The C57B1/6J control group received through oral gavage administration sterilized water (0.1 ml/10 g body weight), once per day for 35 days. ACCU-CHEK advantage blood glucose detection system from Roche was used to determine the blood glucose levels. Glucose tolerance test was performed after 35 days of the indicated treatments following a 3 h of fasting. The blood glucose was measured before (0 min) or 60 min and 120 min after administration of 2.5 g/kg glucose. Area under curve (AUC) was estimated using the formula AUC = 1 / 2(0 h + 1 h) ∗ 0.5 + 1 / 2(1 h + 2 h) ∗ 1.5. To determine insulin levels (right after the 35 day treatment period) the rat/mouse Insulin ELISA Kit from Millipore was used according to the manufacturers' instructions.

No differences between the treatment groups were detected in terms of routine activity of the animals, hair condition, food intake and water intake.

### 2.9 Molecular docking

Docking experiments of honokiol into the hPPARγ ligand binding domain (Protein Data Bank, www.pdb.org, PDB ID: 2VSR [24]) were performed using the CDOCKER module of Discovery Studio 2.5 (www.accelrys.com) with default settings. For the first ligand, the binding site was defined as a 9 Å sphere, centered on the guanidine carbon of Arg288. The binding site for the second ligand was defined as an 8 Å sphere, centered on the sulfur atom of Cys285. Predicted binding poses were visualized and ligand–protein interactions analyzed with LigandScout 3.03 (www.inteligand.com).

### 2.10 Statistical methods and data analysis

Statistical analyses were done with the GraphPad Prism software version 4.03. One-way or two way analysis of variance (ANOVA) with Bonferroni post hoc test was used to determine statistical significance. Nonlinear regression (sigmoidal dose response) was used to calculate the EC_50_ values and maximal fold activation. *K_i_* values of the investigated compounds in the receptor binding assay were calculated with the Cheng–Prusoff equation: (*K_i_*) = IC_50_ / (1 + L / K_D_)), where IC_50_ is the concentration of the competitor that produces 50% displacement of the ligand, L is the concentration of Fluormone™ Pan-PPAR Green used in the assay (5 nM), and K_D_ is the binding constant (2.8 nM) of Fluormone™ Pan-PPAR Green to PPARγ-LBD [25].

## 3 Results

### 3.1 Identification of honokiol as a novel partial PPARγ agonist

**Fig. 1:**
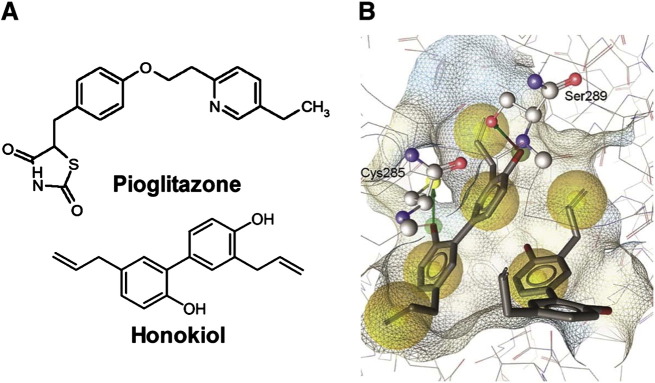
Chemical structures (A) of pioglitazone and honokiol, and honokiol modeled in the PPARγ ligand-binding pocket (B). Yellow spheres mark hydrophobic protein–ligand contacts, red and green arrows hydrogen bonds between honokiol and Cys285 and Ser289, respectively.

**Fig. 2:**
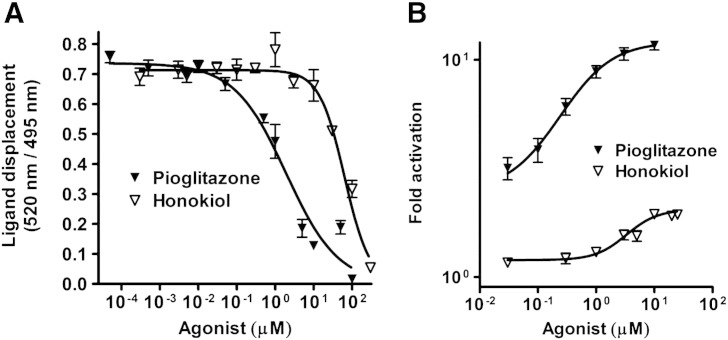
PPARγ receptor binding and luciferase reporter transactivation of honokiol. (A) PPARγ binding of honokiol. Serial dilutions of the honokiol and pioglitazone were prepared in DMSO and then mixed with a buffer solution containing the human PPARγ LBD tagged with GST, a terbium-labeled anti-GST antibody, and fluorescently labeled PPAR agonist. After 1 h of incubation, the ability of the test compounds to bind to the PPARγ LBD and thus displace the fluorescently labeled ligand was estimated from the decrease of the emission ratio 520 nm/495 nm upon excitation at 340 nm. Each data point represents the mean ± standard error of the mean (SEM) from three independent experiments performed in duplicate. (B) Influence of honokiol on human PPARγ-mediated luciferase reporter gene transactivation. HEK-293 cells, transiently transfected with a plasmid encoding full-length human PPARγ, a reporter plasmid containing PPAR-response element coupled to a luciferase reporter, and EGFP as internal control, were stimulated with the indicated concentrations of honokiol and pioglitazone for 18 h. Luciferase activity was normalized to the EGFP-derived fluorescence, and the result was expressed as fold induction compared with the negative control (DMSO vehicle treatment). The data shown are means ± SEM of three independent experiments each performed in quadruplicate.

We aimed to characterize novel partial PPARγ ligands, which can reduce blood sugar levels without inducing weight gain. Using the pharmacophore model collection previously developed [26], we have recently identified several neolignans as representatives of a novel class of partial PPARγ agonists binding to the receptor LBD as dimers [17]. In the present study another structurally similar neolignan, the natural product honokiol (Fig. 1A), was predicted by molecular docking to also bind to PPARγ in a dimeric mode (Fig. 1B). In order to confirm that honokiol indeed binds to PPARγ we performed a competitive FRET-based ligand binding assay with the purified human PPARγ LBD. Honokiol directly bound to the purified human PPARγ LBD in vitro (Fig. 2A; *K_i_* = 22.86 μM), although weaker than pioglitazone (Figs. 1A and 2A; *K_i_* = 0.69 μM), which served as a reference full agonist for all conducted experiments. Honokiol also bound to the LBDs of PPARα and PPARβ/δ but only at considerably (three to four-fold) higher concentrations than required for PPARγ binding (data not shown). In a next step we examined honokiol for its transactivation potential using a PPARγ-driven luciferase reporter gene assay. Honokiol hereby displayed partial PPARγ agonism. It activated the receptor with an EC_50_ value of 3.9 μM and reached maximally two-fold activation compared to 12-fold maximal activation with an EC_50_ value of 0.3 μM by the full agonist pioglitazone in the same system (Fig. 2B).

### 3.2 Honokiol stimulates basal glucose uptake in 3T3 adipocytes

**Fig. 3:**
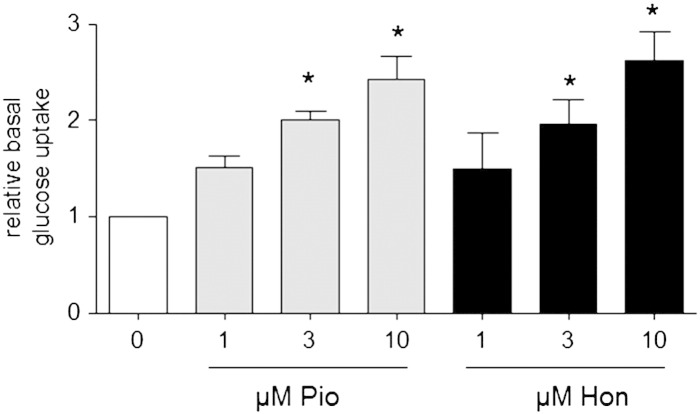
Basal glucose uptake rate upon treatment with honokiol in differentiated adipocytes. Differentiated 3T3-L1 adipocytes were treated with DMSO (solvent control; 0 μM) or different concentrations of pioglitazone (Pio) and honokiol (Hon) (1–10 μM) for 24 h before the basal cellular glucose uptake rate was determined as described in detail in the “Materials and methods” section. The bar graph depicts compiled results of three independent experiments (means ± SEM, *p < 0.05, statistically different to DMSO control group, ANOVA, Bonferroni post test).

In order to assess PPARγ agonism in a functional cell model with endogenous levels of PPARγ, we investigated the potential anti-hyperglycemic activity of honokiol and pioglitazone in mature 3T3-L1 adipocytes. We incubated the cells with different non-toxic concentrations of pioglitazone or honokiol (1–10 μM, supplemental Fig. S5) for 24 h and determined the basal cellular glucose uptake rate. As seen in Fig. 3, both compounds enhanced the cellular glucose uptake in adipocytes in a concentration dependent manner and also to a comparable extent. At a concentration of 10 μM both compounds approximately doubled the amount of ingested glucose compared to vehicle-treated cells.

### 3.3 Honokiol does not induce adipogenic differentiation in vitro

**Fig. 4:**
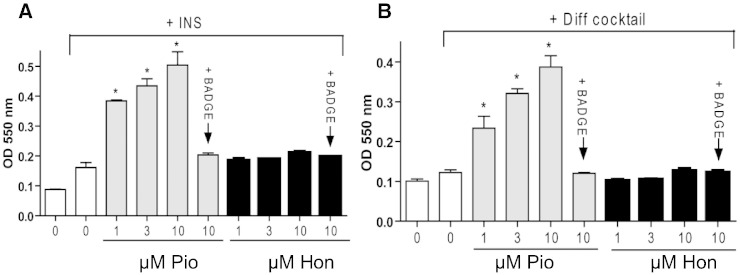
Adipogenic potential of honokiol. 3T3-L1 preadipocytes (A) and mouse embryonic fibroblasts (B) were grown in 12-well plates to superconfluency and induced to differentiate into adipocytes and accumulate lipid in a PPARγ-dependent way as described in detail in the “Materials and methods” section (INS: insulin-containing 3T3-L1 basal differentiation medium, Pio: pioglitazone (0–10 μM), Hon: honokiol (1–10 μM), Diff Cocktail: basal MEF differentiation cocktail containing insulin, IBMX, dexamethasone and bone morphogenic protein 4). BADGE (50 μM) was used as PPARγ antagonist. After staining the cells with the lipophilic dye OilRed O the plates were photographed (see supplemental Fig. S6). Bound dye was solubilized and quantified spectrophotometrically at 550 nm. The bar graphs depict compiled results of four (3T3-L1) or three (MEF) independent experiments (means ± SEM, *p < 0.05, statistically different to “INS” and “Diff cocktail”, respectively; ANOVA, Bonferroni post-test).

We further assessed the adipogenic potential of honokiol and pioglitazone. For this, we chose two different cellular adipogenesis models [27]. In both 3T3-L1 preadipocytes (Fig. 4A) and immortalized mouse embryonic fibroblasts (Fig. 4B) pioglitazone significantly enhanced adipogenic differentiation in a concentration-dependent manner. The induction of differentiation by pioglitazone was PPARγ-dependent and not an off-target result of pioglitazone treatment as it was blunted by co-treatment with the known PPARγ antagonist BADGE. Honokiol, even at the highest test concentration (10 μM), did not lead to a markedly enhanced formation of intracellular lipid droplets above basal levels (see also supplemental Fig. S6).

### 3.4 Honokiol prevents in vivo exacerbation of hyperglycemia and weight gain in the KKAy murine diabetes model

**Fig. 5:**
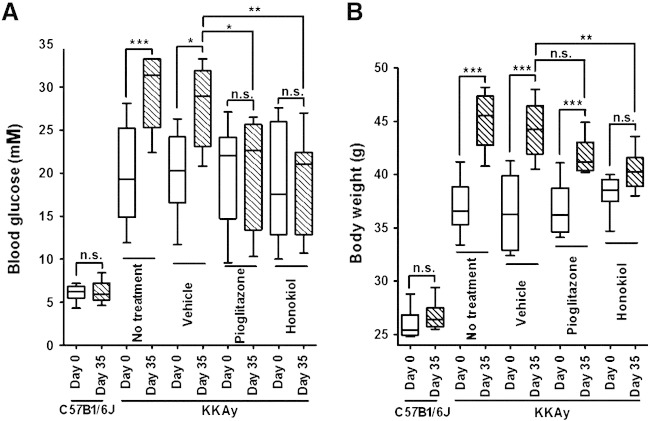
Effect of honokiol on the blood glucose and body weight of diabetic KKAy mice. Blood glucose (A) and body weight (B) of KKAy mice administered orally with honokiol (100 mg/kg/day), pioglitazone (10 mg/kg/day) or vehicle for 35 days is presented (box and whisker plots representing median, upper and lower quartiles, and lowest and highest detected values. ***p < 0.001, **p < 0.01, *p < 0.05 (n = 10, one-way ANOVA/Bonferroni)).

So far we have shown that honokiol binds to PPARγ and is a partial (eliciting submaximal activation) and apparently selective (hyperglycemic but not adipogenic) PPARγ modulator in vitro. We went on to challenge these promising findings with the in vivo situation. For this, we chose diabetic KKAy mice which are characterized by gradually worsening obesity and hyperglycemia. We administered honokiol (100 mg/kg), pioglitazone (10 mg/kg), or vehicle for 35 days to these mice. Whereas both pioglitazone and honokiol successfully prevented exacerbation of hyperglycemia (Fig. 5A), only honokiol also significantly suppressed weight gain (Fig. 5B). Furthermore, honokiol significantly (p < 0.001) improved the glucose tolerance and the insulin levels of treated animals (Table 1). These results fit well with the in vitro experiments outlining honokiol as an efficient non-adipogenic blood glucose lowering agent, although further in vivo experiments, particularly with lower administration doses, are required to better estimate its therapeutic potential.

## 4 Discussion

We report in this study the identification and characterization of the natural product honokiol as a novel non-adipogenic partial PPARγ agonist. Interestingly, honokiol is one of the main bioactive constituents (1–5% content) of the traditional Chinese herbal drug Magnolia bark (Cortex Magnoliae, Hou Po), used to treat metabolic disease among others [28], and may thus contribute to the beneficial properties of this herbal remedy via partial activation of PPARγ.

Honokiol is a representative of the chemical class of neolignans, which we recently identified as novel partial PPARγ agonists binding to the receptor LBD as dimers [17]. Shortly after our findings, magnolol, one of our investigated neolignan-type PPARγ agonists, was shown to also bind to RXRα [29]. The co-crystal structures with PPARγ and RXRα revealed a unique mode of dual PPARγ/RXR agonism by magnolol [29]. In addition to the partial PPARγ agonism that we demonstrate here (Fig. 2A,B), honokiol has recently also been shown to directly bind to RXR [30]. Honokiol therefore conforms to the dual PPARγ/RXR binding mode demonstrated for the structurally similar magnolol [29]. However, whereas all previously described neolignans, including the dual PPARγ/RXR agonist magnolol, induced PPARγ-dependent lipid accumulation in 3T3-L1 adipocytes similar to pioglitazone [17], honokiol did not (Fig. 4) and therefore emerges as superior novel PPARγ modulator. Interestingly, in contrast to our findings Choi et al. [31] reached the conclusion that honokiol did not directly bind to PPARγ and induced adipogenic differentiation in 3T3-L1 preadipocytes. One reason for this discrepancy could be the different ranges of used honokiol concentrations. Whereas Choi et al. tested honokiol up to 30 μM, we have also investigated the effect of the compound at several fold higher concentrations (Fig. 1A). Another relevant consideration is that those authors based the exclusion of honokiol as direct PPARγ-binder on a negative result from a TRAP220/DRIP-2 coactivator peptide recruitment assay [31]. An alternative explanation for that observation could be that the binding of honokiol to PPARγ LBD induces a receptor–ligand complex with a conformation that is not optimal for recruitment of the TRAP220/DRIP-2 coactivator peptide in particular. In support of the latter interpretation, our receptor-binding assay (Fig. 2A) unbiasedly shows that honokiol is able to displace fluorescently labeled PPARγ agonist from the LBD and thus directly binds to the ligand binding pocket of PPARγ. It is well known that different ligands induce receptor–ligand complexes with different conformations, leading to the recruitment of distinct sets of coactivators to PPAR-γ and expression of distinct sets of genes [32,33]. In line, another partial PPARγ agonist, MBX-102/JNJ39659100, was previously demonstrated to be unable to trigger TRAP220 recruitment to PPARγ while retaining antidiabetic properties without inducing weight gain [34]. To carefully investigate a potential pro-adipogenic activity of honokiol we used two different in vitro adipogenesis models. Whereas the full agonist pioglitazone induced a potent PPARγ-dependent adipogenic differentiation in both cellular models, honokiol did not display adipogenic properties (Fig. 4A and B). Moreover, the favorable activity profile observed in vitro was further confirmed in vivo in the diabetic KKAy mouse model in which honokiol not only prevented rising hyperglycemia similar to pioglitazone (Fig. 5A) but also suppressed weight gain (Fig. 5B) and clearly negatively influenced adipogenesis in our experiments. At the current stage, however, it cannot be excluded that proteins other than PPARγ contribute to the beneficial in vivo effects of honokiol. Further support for PPARγ activation by honokiol in vivo may be provided in a recent work from Diano et al. who show that honokiol administration mimics the modulatory effect of rosiglitazone on the activation of hypothalamic neuronal circuits involved in body weight control [35]. However, this observation was then interpreted as a consequence of the ROS scavenging properties of honokiol, and the possibility that the compound could act as a direct activator of PPARγ was not considered by the authors.

Overall, the here identified partial PPARγ activation pattern, in combination with suppression of hyperglycemia and weight gain, makes honokiol an interesting natural product with a good potential to be further explored as pharmaceutical lead or dietary supplement.

## Conflict of interest statement

The authors have no conflict of interest concerning this work.

## Figures and Tables

**Table 1:** Effect of honokiol or pioglitazone treatment on the glucose tolerance of KKAy mice. Glucose tolerance test and blood insulin level determination were performed after 35 days of the indicated treatments following 3 h of fasting. The blood glucose was measured before (0 min) or 60 min and 120 min after administration of 2.5 g/kg glucose. AUC was estimated using the formula AUC = 1 / 2(0 h + 1 h) ∗ 0.5 + 1 / 2(1 h + 2 h) ∗ 1.5. The shown data represent mean ± SD. ***p < 0.001, **p < 0.01, n.s. not significant, compared to the KKAy: vehicle group (ANOVA/Bonferroni).

	Blood glucose (mM)			AUC	Blood insulin (ng/ml)
	0 min	60 min	120 min		
C57B1/6J	6.11 ± 1.03***	6.83 ± 1.02***	6.16 ± 1.08***	12.98 ± 1.60***	0.51 ± 0.15***
KKAy: no treatment	29.57 ± 4.29^n.s.^	30.6 ± 3.02^n.s.^	26.38 ± 4.49^n.s.^	57.80 ± 5.94^n.s.^	33.52 ± 3.34^n.s.^
KKAy: vehicle	27.25 ± 3.25	29.4 ± 2.71	26.93 ± 2.20	56.42 ± 3.03	29.43 ± 6.44
KKAy: pioglitazone	19.54 ± 6.43***	19.96 ± 5.30***	16.64 ± 4.72***	37.34 ± 10.20***	24.39 ± 8.65^n.s.^
KKAy: honokiol	15.59 ± 2.71***	22.94 ± 5.51^⁎⁎^	12.63 ± 3.64^⁎⁎⁎^	36.32 ± 7.41 ^⁎⁎⁎^	15.23 ± 7.71***
