# Supplementary material for: Honokiol: A non-adipogenic PPARγ agonist from nature
Source: Biochim Biophys Acta. 2013 Oct;1830(10):4813–9. doi: 10.1016/j.bbagen.2013.06.021 (PMC3790966; doi:10.1016/j.bbagen.2013.06.021)
Supplement: Supplementary file 1 — Supplementary material. [file mmc1.pdf]

Supplementary methods

Cell viability assays

Differentiated 3T3-L1 adipocytes were exposed to different concentrations (0-30 µM) of pioglitazone and honokiol for 24 hrs before cell viability was determined by two different methods. As readout for cell viability we determined cellular ATP levels using the luminescence-based CellTiterGlo^{R} assay from Promega according to the manufacturer´s instructions. As readout for cytotoxicity of the compounds we assessed membrane integrity and determined the amount of lactate dehydrogenase (LDH) released from the cells using Promega´s CytoTox^{R} non-radioactive cytotoxicity assay.

List of supplementary tables:

Table S1: ^{1}H (300 MHz) and ^{13}C NMR (75 MHz) of honokiol. (CD_3Cl_3, δ in ppm, *J* in Hz).

List of supplementary figures:

Fig. S1: ^{1}H-NMR spectrum (in $CDCl_3$; 300 MHz) of honokiol.

Fig. S2: HSQC spectrum (in CDCl3; 300 MHz/75 MHz) of honokiol.

Fig. S3: HMBC spectrum (in $CDCl_3$; 300 MHz/75 MHz) of honokiol.

Fig. S4: LC-ESI-MS spectrum (neg. mode) of honokiol.

Fig. S5: Influence of pioglitazone and honokiol on viability of 3T3-L1 adipocytes

Fig. S6: Adipogenic potential of the PPARγ agonists pioglitazone and honokiol.

Table S1: ^{1}H (300 MHz) and ^{13}C NMR (75 MHz) of honokiol. (CD_3Cl_3, δ in ppm, *J* in Hz)

Position	δ_H mult. (*J*)	δ_C mult.
1	-	127.8 *s*
2	-	150.8 *s*
3	6.90 *d* (8.1)	115.5 *d*
4	7.05 *dd* (2.1, 8.2)	128.8 *d*
5	-	132.2 *s*
6	7.03 *d* (2.2)	130.2 *d*
7	3.36 *d* (6.7)	39.4 *t*
8	5.98 *m*	137.8 *d*
9	5.08 *m*	115.5 *t*
1’	-	129.5 s
2’	7.23 *dd* (2.1, 7.2)	128.5 *d*
3’	6.91 *d* (8.1)	116.6 *d*
4’	-	153.9 *s*
5’	-	126.4 *s*
6’	7.22 br *s*	131.1 *d*
7’	3.47 *d* (6.1)	35.1 *t*
8’	6.04 *m*	136.0 *d*
9’	5.21 *m*	116.9 *t*

Fig. S1: ^{1}H-NMR spectrum (in $CDCl_3$; 300 MHz) of honokiol.

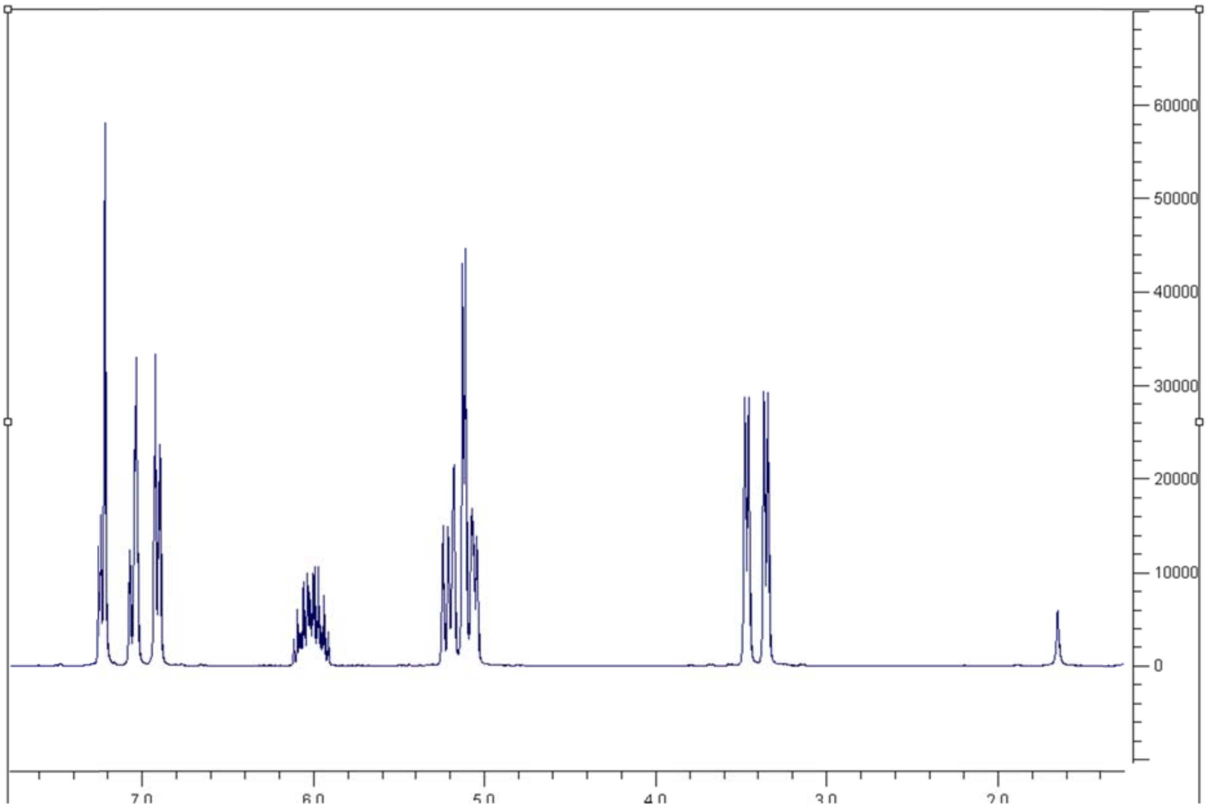

Fig. S2: HSQC spectrum (in $CDCl_3$; 300 MHz/75 MHz) of honokiol.

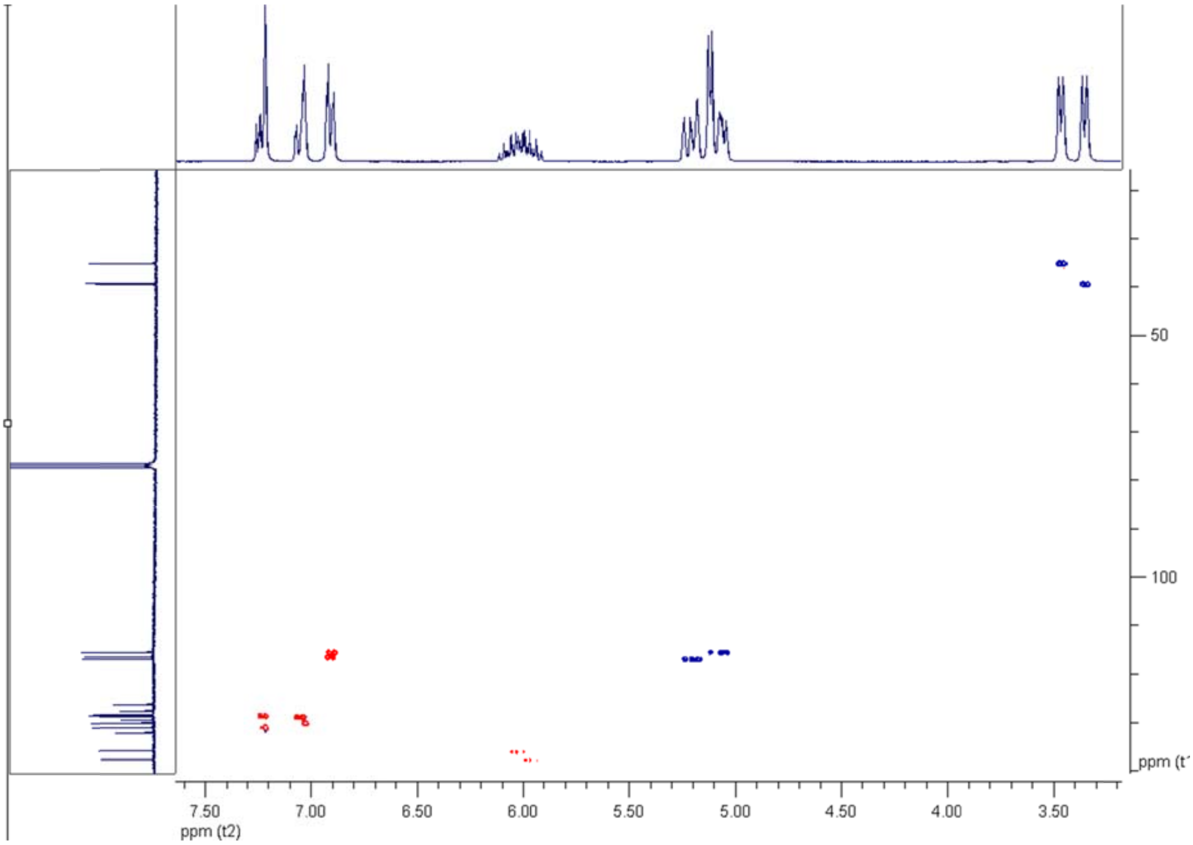

Fig. S3: HMBC spectrum (in $CDCl_3$; 300 MHz/75 MHz) of honokiol.

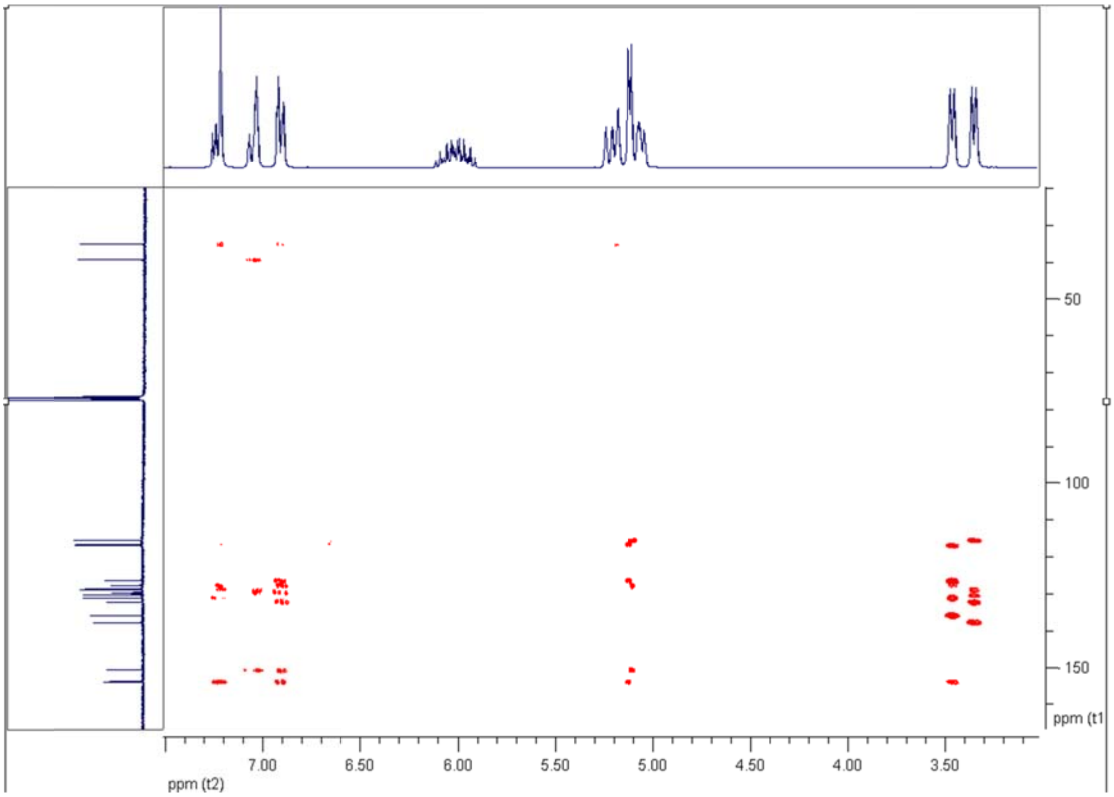

Fig. S4: LC-ESI-MS spectrum (neg. mode) of honokiol.

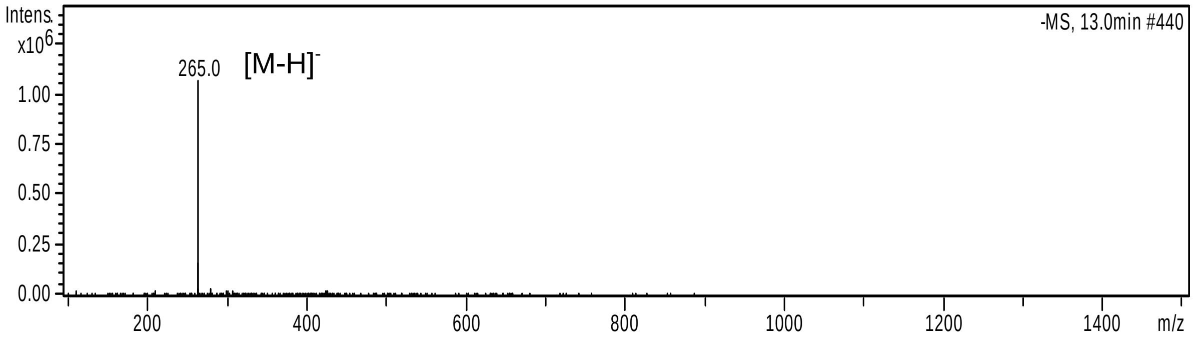

Fig. S5: Influence of pioglitazone and honokiol on viability of 3T3-L1 adipocytes

A

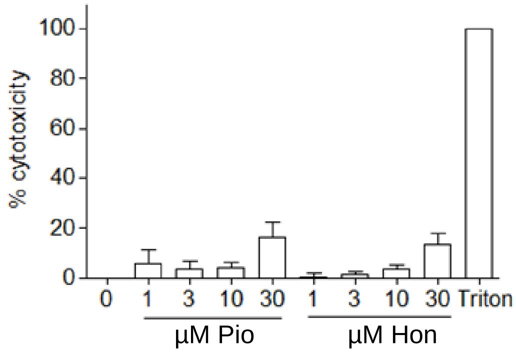

B

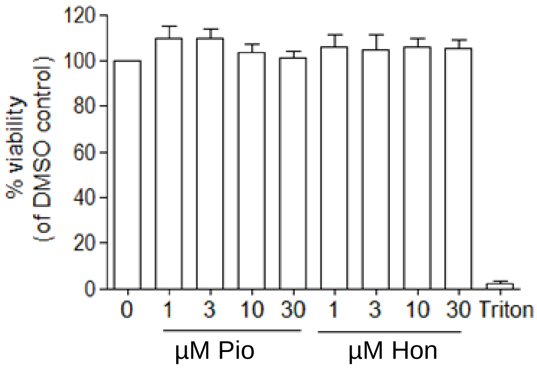

Differentiated 3T3-L1 adipocytes were exposed to pioglitazone (Pio) and Honokiol (Hon), respectively, in a concentration range from 1 µM to 30 µM for 24 hrs before (**A**) the cellular release of LDH (1% Triton served as positive control (100 % cytotoxicity)) and (**B**) the cellular level of ATP as readout for cell viability (DMSO treated cells: 100 % viability) were determined. The bar graph depicts compiled data from three independent experiments performed in duplicate.

➔ Between 0 and 30 µM, neither Pio nor Hon exerts a marked negative impact on cell viability.

Fig. S6: Adipogenic potential of the PPARγ agonists pioglitazone and honokiol.

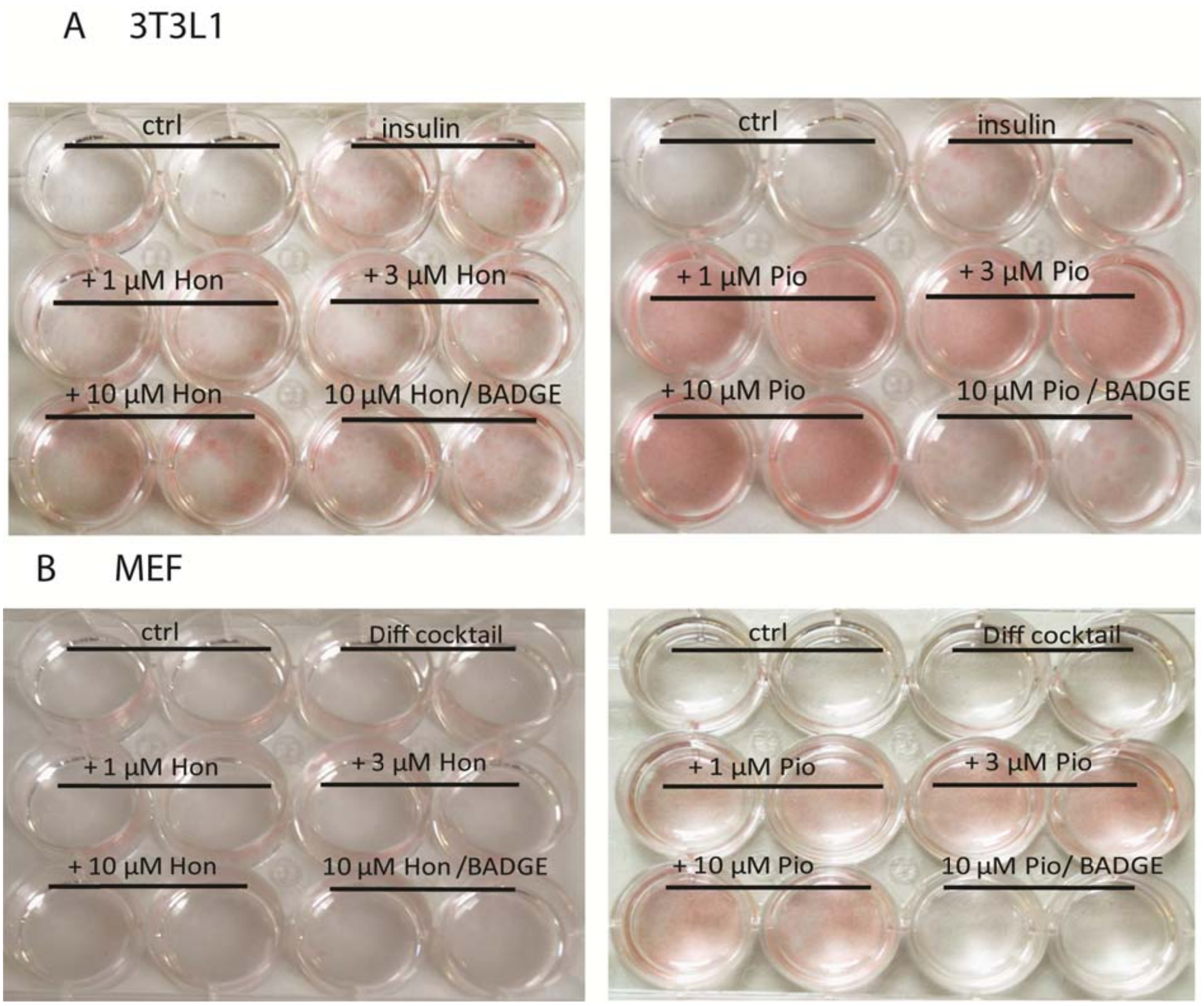

3T3L1 preadipocytes (**A**) and mouse embryonal fibroblasts (**B**) were grown in 12-well plates to superconfluency and induced to differentiate into adipocytes and accumulate lipid in a PPAR gamma dependent way as described in detail in Material and Methods (INS: insulin-containing 3T3-L1 basal differentiation medium, Pio: pioglitazone (1-10 µM), Hon: honokiol (1-10 µM), Diff Cocktail: basal MEF differentiation cocktail containing insulin, isobutylmethylxanthine, dexamethasone and bone morphogenic protein 4). After staining the cells with the lipophilic dye OilRed O the plates were photographed. Photos of one representative experiment (out of three) are shown.
